# ALVEOLAR RECRUITMENT MANEUVERS FOR CHILDREN WITH CANCER AND ACUTE RESPIRATORY DISTRESS SYNDROME: A FEASIBILITY STUDY

**DOI:** 10.1590/1984-0462/2021/39/2019275

**Published:** 2021-01-11

**Authors:** Marcela Salvador Galassi, Rodrigo Genaro Arduini, Orlei Ribeiro de Araújo, Rosa Masssa Kikuchi Sousa, Antonio Sergio Petrilli, Dafne Cardoso Bourguignon da Silva

**Affiliations:** a Universidade Federal de São Paulo, São Paulo, SP, Brazil.

**Keywords:** Respiratory insufficiency, Neoplasms, Acute respiratory distress syndrome, Child, Insuficiência respiratória, Neoplasias, Síndrome do desconforto respiratório agudo, Criança

## Abstract

**Objective:**

Acute respiratory distress syndrome (ARDS) can be a devastating condition in children with cancer and alveolar recruitment maneuvers (ARMs) can theoretically improve oxygenation and survival. The study aimed to assess the feasibility of ARMs in critically ill children with cancer and ARDS.

**Methods:**

We retrospectively analyzed 31 maneuvers in a series of 12 patients (median age of 8.9 years) with solid tumors (n=4), lymphomas (n=2), acute lymphoblastic leukemia (n=2), and acute myeloid leukemia (n=4). Patients received positive end-expiratory pressure from 25 up to 40 cmH_2_0, with a delta pressure of 15 cmH_2_O for 60 seconds. We assessed blood gases pre- and post-maneuvers, as well as ventilation parameters, vital signs, hemoglobin, clinical signs of pulmonary bleeding, and radiological signs of barotrauma. Pre- and post-values were compared by the Wilcoxon test.

**Results:**

Median platelet count was 53,200/mm^3^. Post-maneuvers, mean arterial pressure decreased more than 20% in two patients, and four needed an increase in vasoactive drugs. Hemoglobin levels remained stable 24 hours after ARMs, and signs of pneumothorax, pneumomediastinum, or subcutaneous emphysema were absent. Fraction of inspired oxygen decreased significantly after ARMs (FiO_2_; p=0.003). Oxygen partial pressure (PaO_2_)/FiO_2_ ratio increased significantly (p=0.0002), and the oxygenation index was reduced (p=0.01), but all these improvements were transient. Recruited patients’ 28-day mortality was 58%.

**Conclusions:**

ARMs, although feasible in the context of thrombocytopenia, lead only to transient improvements, and can cause significant hemodynamic instability.

## RESUMO

**Objetivo:**

A síndrome do desconforto respiratório agudo (SDRA) pode ser uma condição devastadora em crianças com câncer e as manobras de recrutamento alveolar (MRA) podem melhorar a oxigenação e a sobrevida. O objetivo foi avaliar a viabilidade das MRA em crianças gravemente doentes com câncer e SDRA.

**Métodos:**

Analisamos retrospectivamente 31 manobras em 12 pacientes (idade mediana de 8,9 anos), com tumores sólidos (n=4), linfomas (n=2) e leucemias linfoide (n=2) e mieloide agudas (n=4). Os pacientes receberam pressão expiratória final positiva de 25 a 40 cmH_2_0, com delta de pressão de 15 cmH_2_O por 60 segundos. Gasometrias foram analisadas pré e pós-manobras, bem como os parâmetros de ventilação, sinais vitais, hemoglobina, sinais clínicos de sangramento pulmonar e sinais radiológicos de barotrauma. Valores foram comparados com o teste de Wilcoxon.

**Resultados:**

A contagem mediana de plaquetas era de 53.200/mm^3^. Após as manobras, em dois pacientes, a pressão arterial média declinou mais de 20%, e quatro necessitaram de aumento de drogas vasoativas. A hemoglobina permaneceu estável 24 horas após a MRA, sem sinais de pneumotórax, pneumomediastino ou enfisema subcutâneo. Houve diminuição significativa nas frações inspiradas de oxigênio (FiO_2_; p=0,003). A relação pressão arterial de oxigênio (PaO_2_)/FiO_2_ aumentou (p=0,002), e o índice de oxigenação caiu (p=0,01), mas essas melhoras foram transitórias. A mortalidade em 28 dias foi de 58%.

**Conclusões:**

As MRA, embora viáveis no contexto da trombocitopenia, levam apenas a melhorias transitórias e podem causar instabilidade hemodinâmica significativa.

## INTRODUCTION

Acute respiratory failure is a frequent condition in children with cancer admitted to the Intensive Care Unit (ICU). Those who develop acute respiratory distress syndrome (ARDS) and need mechanical ventilation (MV) have a poor prognosis.^1^ Twenty-eight-day mortality can be as high as 58% in patients with neoplasms, septic shock, and ARDS.^2^ Studies performing lung computed tomography (CT) have shown that ARDS presents multiple gravity-dependent atelectasis areas, which are prone to opening with alveolar recruitment maneuvers (ARMs).^3^ Extension of the recruitment area is influenced by the time elapsed since ARDS onset, and ARMs have their best efficacy the earlier they are initiated due to the development of fibrosis in the affected lung in later stages of the disease. In lungs progressing with increased resistance, high positive end-expiratory pressure (PEEP) settings during ARMs can lead to complications, particularly hemodynamic impairment.^4^

Few studies have analyzed alveolar recruitment in the pediatric population and none within the cancer subpopulation. The present study aimed to evaluate the feasibility of ARMs in critically ill pediatric cancer patients who developed acute respiratory failure due to ARDS.

## METHOD

This is a retrospective study approved by the Ethics Committee of Universidade Federal de São Paulo (UNIFESP) (protocol no. 12802/2009), and the informed consent form was waived. We evaluated all data on electronic medical records from patients aged 0 to 17 years, admitted to our 11-bed oncologic ICU from January 1, 2010 to December 31, 2011, with an ARDS diagnosis according to the American-European Consensus Conference.^5^ Exclusion criteria were: severe hemodynamic instability at the moment of ARMs (need for vasopressors - epinephrine or norepinephrine - greater than 0.05 mcg/kg/min or progressive titrating doses 2 hours prior to intervention); extreme agitation/anxiety; untreated pneumothorax; signs of alveolar or digestive hemorrhage; bronchopleural fistulae; or intracranial hypertension.^6^ ^,^ ^7^

Following an institutional protocol, all patients were under continuous sedation and received neuromuscular blockade (rocuronium) before ARMs. Patients had to be at the first week of disease. ARMs were carried out in pressure-controlled ventilation mode, with a delta pressure [i.e., peak inspiratory pressure (PIP) minus PEEP] of 15 cmH_2_O. The initial PEEP value varied from 25 to 40 cmH_2_O. It was kept for 60 seconds if no hemodynamic instability (that is, bradycardia or decrease in blood pressure >20%) was observed. ARMs could be performed three times if oxygenation was not sustained. For example, children with less than 20 kg were submitted to an initial ARM at a PEEP of 25 cmH_2_O. ARM could be repeated at a PEEP of 30, with one last ARM at a PEEP of 35 cmH_2_O. Children with more than 20 kg were submitted to the first ARM at a PEEP of 30 cmH_2_O, the second (if necessary) at a PEEP of 35, and the last one at a PEEP of 40, always with a delta pressure of 15 cmH_2_O.

**Figure 1:**
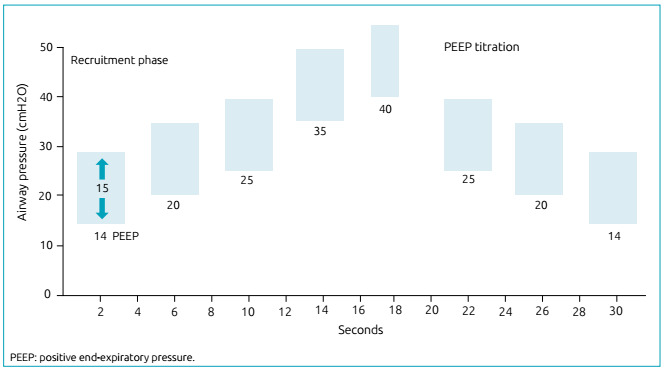
Positive end-expiratory pressure escalation and titration during the alveolar recruitment maneuver.

At the start of the titration phase, PEEP was reduced in decrements of 2-3 points, with less than ten seconds for each step, until oxygen saturation reached values lower than 92%. This was considered the desaturation point. The maneuver was performed again, and PEEP was set 2 cmH_2_O above this desaturation point (Figure 1).^8^ ^,^ ^9^

We analyzed blood gases before and after ARMs, as well as MV parameters, heart rate, mean arterial pressure (MAP), pulse oximetry, and vasopressor doses. Chest radiographs were reviewed to detect pneumothorax, pneumomediastinum, and pulmonary interstitial emphysema. Hemoglobin levels and subcutaneous emphysema were also monitored for 24 hours after ARMs. Oxygenation index (OI) was calculated according to Ortiz et al.: OI = fraction of inspired oxygen (FiO_2_) × mean airway pressure (mPaw) / oxygen partial pressure in arterial blood (PaO_2_).^10^ Dynamic compliance was calculated as: tidal volume / (PIP - PEEP).^11^

We used the Wilcoxon signed-rank test to compare pre- and post-maneuver values. The Monte Carlo method determined the 99% confidence intervals, with resampling of at least 1000 tables due to the small sample size. Statistical significance was set at p<0.05. All statistical analyses were performed using the SPSS software, version 20.0 (IBM Corp., Armonk, NY, USA).

## RESULTS

We analyzed 31 maneuvers performed in 12 patients, with a median age of 8.9 years (ranging from 9 months to 16 years). Table 1 presents the diagnoses: four patients had solid tumors, and eight had hematologic malignancies (one of them had undergone bone marrow transplantation). Maneuvers were performed in patients with a median of 53,200 platelets/mm^3^ (P25-75 = 32,200-122,530).

**Table 1:** Cancer and acute respiratory distress syndrome associated diagnoses in the 12 patients. Disease times are counted until the moment of the recruitment maneuvers.

Patient	Underlying disease	ARDS associated diagnosis	Age (years)	Time from cancer diagnosis (months)	Time from ARDS diagnosis (days)	Death
1	Bulbo-medullary glioma	Aspiration pneumonia / septic shock	8.6	2	2.4	No
2	Optic glioma	Febrile neutropenia / septic shock	1.1	1	3	No
3	AML	Septic shock	1.2	8	2.5	Yes
4	Hepatoblastoma	Pneumonia / septic shock	2	17	3.2	Yes
5	Hodgkin’s lymphoma	Pneumonia / septic shock	16.3	10	1.8	Yes
6	AML	Febrile neutropenia / septic shock	10.5	4	3	Yes
7	ALL	Febrile neutropenia / septic shock	10.9	10	2	Yes
8	ALL	Septic shock	15	72	1.8	No
9	Non-Hodgkin’s lymphoma	Septic shock	9	7	2.7	Yes
10	Fibrosarcoma (jaw)	Post-cardiac arrest syndrome	0.7	1	2.6	No
11	AML	Retinoic acid syndrome	11	8	1.9	No
12	AML / hematopoietic stem-cell transplantation	Septic shock	16	2	2.4	Yes

ARDS: acute respiratory distress syndrome; AML: acute myeloid leukemia; ALL: acute lymphoblastic leukemia.

During ARMs, the minimum PEEP was 25 and the maximum, 40 cmH_2_O [interquartile range (IQR) 27-75: 35-40 cmH_2_O]. Maximum PIP was 55 cmH_2_O (IQR: 50-55 cmH_2_O), and the mean respiratory rate was 4.7 breaths per minute [standard deviation (SD): 3].

We found no significant changes in heart rate during ARM (mean pre-ARM: 136.7 beats per minute, SD: 26.5; post-ARM: 137.3, SD: 20.8). During seven maneuvers performed in five patients, MAP had a decline greater than 5% (12.9 mmHg on average, SD: 8.1), leading to an increase in rates of infusion of vasoactive drugs in four of them (33%). In two patients (16.6%), the reduction in MAP was greater than 20%. During six ARMs performed in two patients (16.6%), we identified a transient increase in MAP greater than 5% (mean: 23.7 mmHg, SD: 27.5). Mean MAP was 79 mmHg (SD: 16.8) before and 80.2 mmHg (SD: 18.5) after alveolar recruitment (p=0.93).

None of the children experienced a decrease in hemoglobin levels in the 24 h following ARMs. No major air leaks were detected, such as pneumothorax, pneumomediastinum, and/or subcutaneous emphysema.

FiO_2_ and OI significantly decreased after ARMs (p=0.003 and p=0.01, respectively); the PaO_2_/FiO_2_ ratio had a statistically significant increase (p=0.0002). These improvements in oxygenation were not sustained for more than 2 hours after ARMs, as oxygen saturation declined, and FiO_2_ increased again. All other blood gas parameters analyzed (pH, carbon dioxide partial pressure, bicarbonate, oxygen saturation) showed no signs of significant improvement post-maneuvers. Dynamic lung compliance was not affected either.

**Figure 2:**
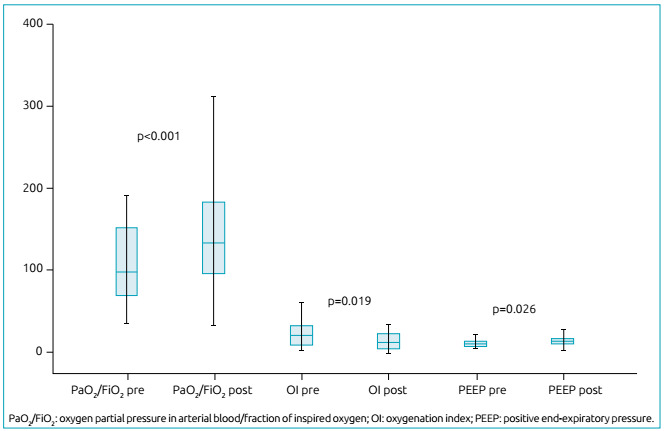
Boxplots of the values for the oxygen partial pressure/fraction of inspired oxygen ratio, oxygenation index, and positive end-expiratory pressure, pre- and post-recruitment maneuver.

PEEP levels remained high after ARMs (p=0.02). Other ventilatory parameters (inspiratory pressure, mPaw, inspiratory time, and tidal volume) showed no significant differences. Table 2 describes MV parameter values and their respective p values. Figure 2 shows the boxplots of significant changes.

**Table 2:** Ventilatory parameters and blood gas values before and after recruitment maneuvers.

	Mean	SD	p-value*	99%CI for p-value**
Peak pressure (cmH_2_0)				
Before	32.6	8.8	0.630	0.630-0.650
After	33.6	10.5		
PEEP (cmH_2_0)				
Before	14.6	5.2	0.026	0.021-0.029
After	17.3	6.1		
Mean pressure (cmH_2_0)				
Before	22.3	7.7	0.270	0.270-0.290
After	20.8	6.4		
Tidal volume (mL/kg)				
Before	188.1	119.9	0.090	0.084-0.099
After	176.3	107.3		
Dynamic compliance (mL/cmH_2_0)				
Before	11.0	7.7	0.840	0.850-0.870
After	11.1	6.6		
FiO_2_				
Before	0.8	0.2	0.003	0.001-0.003
After	0.6	0.2		
pH				
Before	7.3	0.1	0.180	0.170-0.190
After	7.3	0.1		
Bicarbonate (mmol/L)				
Before	26.3	9.7	0.059	0.052-0.064
After	23.9	7.2		
O_2_ saturation (%)				
Before	91.4	6.6	0.580	0.570-0.590
After	92.0	6.3		
PaCO_2_ (mmHg)				
Before	56.6	21.9	0.190	0.180-0.200
After	59.5	18.2		
PaO_2_ (mmHg)				
Before	73	18.8	0.320	0.330-0.350
After	77.6	7.2		
OI				
Before	25.8	16.4	0.019	0.014-0.021
After	21.4	19.6		
PaO_2_/FiO_2_				
Before	107.1	48.4	0.000	0.000-0.000
After	150.3	72.4		

SD: standard deviation; CI: confidence interval; *Wilcoxon test; **Monte Carlo method; PEEP: positive end-expiratory pressure; FiO_2_: fraction of inspired oxygen; PaCO_2_: carbon dioxide partial pressure in arterial blood; PaO_2_: oxygen partial pressure in arterial blood; OI: oxygenation index.

We also analyzed the first ARM of each patient. Considering only these 12 ARMs, we found significant differences in FiO_2_ pre- and post-maneuvers (pre: 0.80±0.24; post: 0.58±0.22; p=0.05, according to the Wilcoxon test) and also in PaO_2_/FiO_2_ ratios pre- and post-maneuvers (pre: 95.3±49.3 and post: 158.5±72.7; p=0.03). Unfortunately, we could not perform further analyses of these first ARMs due to the small sample size.

## DISCUSSION

In our small group of critically ill patients, recruitment maneuvers produced some degree of improvement in oxygenation parameters, but these improvements were evanescent. The fact that one-third of patients needed more vasoactive drugs is also concerning. The literature has no other studies on ARMs in this population, so we cannot compare results.

Pediatric cancer patients who develop ARDS are extremely ill and have high mortality rates. Ben-Abraham et al. studied 17 children with ARDS and hematologic malignancies under MV; 11 of them died (64.7%).^1^ Another study reported 29 children with cancer and sepsis-related ARDS: only 5 patients survived for more than 60 days. Among them, 31% died as a direct consequence of refractory hypoxemia, and the remaining patients died of multiple organ failure and catecholamine-refractory shock.^2^ ARDS mortality rates in this subpopulation remain unacceptably high, and no effective therapy, including pharmacological agents,^12^ has been reported up to the present day.

Recruitment maneuvers can improve hypoxemia, but their use in critically ill children with cancer causes concerns due to frequent thrombocytopenia and the risk of pulmonary hemorrhage. The best method for performing ARMs is yet to be defined.^13^ The fixed delta pressure method used here seems to allow more hemodynamic stability compared with the sequential method.^8^ ^,^ ^14^ Slowly decreasing PEEP also seems to maintain alveolar stability for longer.^15^ ^,^ ^16^ Studies on adults have demonstrated that optimizing PEEP after ARMs is essential to improve oxygenation,^16^ which was achieved in our subjects by setting PEEP above the desaturation point, presupposing that this point would correspond to the partial alveolar collapse. Boriosi et al.^17^ identified an improvement in the PaO_2_/FiO_2_ ratio that could last up to 12 hours after the maneuvers. In our study, however, improvement lasted for no more than 2 hours, showing the transient efficacy of ARMs, also registered by Kheir et al.^18^

No significant changes were found regarding the patients’ heart rates. Cruces et al. pointed out hypotension as a common side effect of ARM, detecting MAP reductions of 9.2%.^19^ In our study, MAP declines led to increased infusion of vasopressors in four patients (33.3%), highlighting the extreme care needed when performing ARMs. No bleeding or airway complications were noted in the subsequent 24-hour period.

As ARMs improve oxygen saturation during a short period, healthcare providers might feel tempted to perform them frequently, but no data available from controlled studies allow strong conclusions on their efficacy, safety, and long-term consequences in children.^20^

The main limitation of our study is having a sample too small to determine all aspects of ARM safety. Nonetheless, it was performed as a pilot study to assess the feasibility of ARMs in these extremely ill patients.

A recent large, well-designed, adult clinical trial resulted not only in limited success but in higher 28-day mortality in the ARM group;^21^ maximum ARM could even be associated with cardiac arrest. Interestingly, 28-day mortality was higher in this group than in the control group (55.3% vs. 49.3%), but still lower than in the cancer and ARDS population.^21^ Therefore, assessing actual evidence, ARM should be reserved for patients suffering from refractory hypoxemia, already under potentially non-protective high inspiratory or peak pressures and FiO_2_, and not as a routine therapy in ICUs.

In conclusion, ARMs, although viable, have restrictions regarding their effectiveness in children with cancer and ARDS, with transient improvements in oxygenation, but no improvements in lung compliance. Despite the lack of bleeding-related complications, even in the context of thrombocytopenia, hemodynamic instability seems to be a major concern.
